# The genus *Braunsia* Kriechbaumer, 1894 from China with description of two new species (Hymenoptera, Braconidae, Agathidinae)

**DOI:** 10.3897/zookeys.705.14717

**Published:** 2017-10-03

**Authors:** Pu Tang, Cornelis van Achterberg, Xue-Xin Chen

**Affiliations:** 1 State Key Laboratory of Rice Biology, Institute of Insect Sciences, Zhejiang University, Hangzhou 310029, China; 2 Shaanxi Key Laboratory for Animal Conservation / Key Laboratory of Resource Biology and Biotechnology in Western China, College of Life Sciences, Northwest University, Xi’an, Shaanxi 710069, China; 3 Department of Terrestrial Zoology, Naturalis Biodiversity Center, Postbus 9517, 2300 RA Leiden, The Netherlands

**Keywords:** Agathidinae, *Braunsia*, China, key, new record, new species, taxonomy

## Abstract

The species of *Braunsia* Kriechbaumer, 1894 (Hymenoptera, Braconidae, Doryctinae) from China are revised and ten species are recognized. Two new species, *B. guangdongensis* **sp. n.** and *B. shenyangensis* **sp. n.**, are described and illustrated. *B. fumipennis* (Cameron, 1899), *B. pilosa* Belokobylskij, 1986, *B. postfurcalis* Watanabe, 1937, and *B. smithii* (Dalla Torre, 1898), are recorded from China for the first time. A key to the Chinese species of the genus *Braunsia* is provided.

## Introduction

*Braunsia* Kriechbaumer, 1894, is a medium-sized genus of the subfamily Agathidinae (Braconidae) mostly distributed in the Oriental and Afrotropical regions (Shenefelt 1970; Bhat and Gupta 1977; Yu et al. 2017). Sharkey et al. (2006) treated four nominal genera, *Metriosoma* Szépligeti, 1902, *Lissagathis* Cameron, 1911, *Laccagathis* Watanabe, 1934, and *Pholeocephala* van Achterberg, 1988, as synonyms of *Braunsia* s.l. However, *Metriosoma* (= *Lissagathis*) and *Laccagathis* form a separate group because of the absence of notauli and precoxal sulcus combined with a shallow or nearly flat frons behind the antennal socket. *Laccagathis* has the pronotum emarginate medio-anteriorly combined with a shallowly impressed frons. Therefore, we provisionally retain *Laccagathis* as a separate genus because of the apomorphous character states (including the absence of a lateral carina anteriorly on the lateral lobes of the mesoscutum). Hence, we exclude the only species known from China (*Laccagathis formosana* Watanabe, 1934, reported from Taiwan and Zhejiang) in this paper (Chen and Yang 2006; Chou and Sharkey 1989). *Metriosoma* differs also from *Laccagathis* by the presence of a deep antescutal depression. The remainder of *Braunsia* is united by the deeply depressed frons behind the antennal socket. *Pholeocephala* differs by the protuberance on the stemmaticum and a pair of converging grooves medially on the mesoscutum (both are absent in *Braunsia* s.s.).

Chou and Sharkey (1989) recorded two species of *Braunsia* from Taiwan, viz., *B. bipunctata* Enderlein, 1906, and *B. longicoxa* Bhat & Gupta, 1977. Chen and Yang (2006) proposed a new species (*Braunsia pappi* Chen & Yang, 2006) and reviewed the Chinese species, but they overlooked the two species (*B. antefurcalis* Watanabe, 1937, and *B. matsumurai* Watanabe, 1937) recorded by He et al. (2001) for China. In total, five species of *Braunsia* were actually known from China prior to our study.

During our study of Chinese Agathidinae, we discovered ten species of *Braunsia* (of which only three were known from China before), *B. antefurcalis*, *B. fumipennis* (Cameron, 1899), *B. guangdongensis* sp. n., *B. longicoxa*, *B. matsumurai*, *B. pappi*, *B. pilosa* Belokobylskij, 1986, *B. postfurcalis* Watanabe, *B. shenyangensis* sp. n. and *B. smithii* (Dalla Torre, 1898). In this paper both new species are described and illustrated and a key to the Chinese species of *Braunsia* is provided. The problematic variation of *B. bipunctata* Enderlein is discussed.

## Materials and methods

This study is based on specimens preserved in the Parasitic Hymenoptera Collection of Institute of Insect Sciences, Zhejiang University, Hangzhou, China (**ZJUH**), Institute of Zoology, Chinese Academy of Sciences, Beijing, China (**IZCAS**), Shanghai Entomological Museum, Chinese Academy of Sciences, Shanghai, China (**SEMS**), the Entomological Museum of the China Agricultural University, Beijing, China (**CAU**) and the Naturalis Biodiversity Center collection, Leiden, The Netherlands (**RMNH**).

The terminology and measurements used follow van Achterberg (1993). All descriptions and measurements were made under a Zeiss Stemi 2000-C microscope; figures were made by a digital camera (Q-Imaging, Micropublisher, 3.3 RTV) attached to a stereomicroscope (Leica MZ APO, Germany) and Auto-Montage Pro version 5.0 software. Type specimens are deposited in the Parasitic Hymenoptera Collection of the Zhejiang University, Hangzhou, China (ZJUH).

### Key to Chinese species of the genus *Braunsia* Kriechbaumer

**Table:** 

1	Vein cu-a of fore wing postfurcal or interstitial (Figs 39, 54, 62); ovipositor sheath slightly or not widened (Figs 35, 51); pterostigma light brown or yellow (Figs 39, 54, 62); malar space similarly coloured as head, and if paler, then not or hardly contrasting with surrounding colour (Figs 41, 56, 65)	**2**
–	Vein cu-a of fore wing antefurcal (Figs 6, 14, 22, 31, 46); ovipositor sheath ribbon-shaped widened (Figs 1, 10, 19, 28, 42); pterostigma dark brown or black (Figs 6, 14, 22, 31, 46); malar space ivory and distinctly contrasting with surrounding colour (Figs 5, 15, 21, 30, 44)	**6**
2	Length of first tergite 2.8–3.0 times its apical width (Fig. 58); first tergite almost entirely smooth (Fig. 58); length of second tergite 1.7 times its width (Fig. 58); ovipositor sheath almost as long as body (Fig. 51); fore wing without an isolated stigmal spot (Fig. 54)	***B. postfurcalis* Watanabe**
–	Length of first tergite 1.8–2.0 times its apical width (Figs 40, 67); first tergite largely longitudinally carinate (Figs 40, 67); length of second tergite 1.2 times its width (Figs 40, 67); ovipositor sheath distinctly shorter than body (Figs 42, 60); fore wing with an isolated stigmal spot (Fig. 62) or with a large dark brown area below parastigma (Fig. 46)	**3**
3	Propodeum with a closed areola; hind leg yellowish brown (Fig. 35); vein cu-a of fore wing distinctly postfurcal (Fig. 39); stigmal spot included in a dark brown area below parastigma reaching at least middle of fore wing (Fig. 39)	**4**
–	Propodeum without a closed areola (Fig. 68); hind leg black (Fig. 66); vein cu-a of fore wing almost interstitial (Fig. 62); fore wing with a small isolated stigmal spot (Fig. 62)	***B. shenyangensis* sp. n.**
4	Vein 1-R1 of fore wing yellowish, similar to colour of pterostigma; dark brown area below parastigma up to middle of fore wing	***B. smithii* (Dalla Torre)**
–	Vein 1-R1 of fore wing dark brown, darker than yellowish pterostigma (Fig. 39); dark brown area below parastigma nearly up to posterior border of fore wing (Fig. 39)	**5**
5	Basal half of first tergite with distinct striae (Fig. 40); hind tibia brownish yellow (Fig. 35); tegulae and mesoscutum with same colour (Fig. 38)	***B. matsumurai* Watanabe**
–	Basal half of first tergite smooth; hind tibia whitish yellow basally, contrasting with brownish yellow remainder of hind tibia; tegulae whitish yellow, contrasting with brownish yellow mesoscutum	***B. pappi* Chen & Yang**
6	Antenna, hind coxa and hind femur black (Figs 10, 42)	**7**
–	Antenna, hind coxa and hind femur yellowish brown (Figs 1, 19, 28)	**8**
7	Hind tibia black (Fig. 18); mesosoma largely yellowish brown (Figs 12, 16); length of first tergite 3.3 times its apical width (Fig. 17); apical half of first tergite more or less striate (Fig. 17)	***B. fumipennis* (Cameron)**
–	Hind tibia brown (Fig. 50); mesosoma black (Figs 45, 49); length of first tergite 2.3 times its apical width (Fig. 48); apical half of first tergite smooth (Fig. 48)	***B. pilosa* Belokobylskij**
8	Length of first tergite 4.2–5.3 times its apical width (Fig. 34); wing membrane dark brown, but apical third infuscate (Fig. 31); length of second tergite 2.2–2.3 times its apical width (Fig. 34)	***B. longicoxa* Bhat & Gupta**
–	Length of first tergite 2.7–3.8 times its apical width (Figs 8, 26); wing membrane evenly dark brown (Figs 6, 22); length of second tergite 1.5–1.8 times its apical width (Figs 8, 26)	**9**
9	Length of first tergite 2.7–2.8 times its apical width (Fig. 26); length of hind femur 5.2–5.3 times as long as wide (Fig. 27); area below face and clypeus ivory (Fig. 24)	***B. guangdongensis* sp. n.**
–	Length of first tergite 3.2–3.6 times its apical width (Fig. 8); length of hind femur 5.6–6.2 times as long as wide (Fig. 9); area below face and clypeus black or dark brown (Fig. 3)	***B. antefurcalis* Watanabe**

#### Braunsia antefurcalis

Taxon classificationAnimaliaHymenopteraBraconidae

Watanabe, 1937

Figs 1–9

Braunsia antefurcalis Watanabe, 1937: 90; Shenefelt 1970: 370; Belokobylskij 1989: 67; Sharkey 1996: 59; 1998: 529; He et al. 2001: 373.
Braunsia romani Shestakov, 1940: 12; Shenefelt 1970: 375 (syn. by Belokobylskij 1989).
Braunsia graciliventris Belokobylskij, 1989: 70 (syn. by Sharkey 1996).

##### Material examined.

China (ZJUH). Zhejiang prov.: 5♀♀4♂♂, Fengyangshan, 11.VII.1984, Shen Lirong, Nos. 843301, 843302, 843303, 843305, 843306, 843307, 843308, 843309, 843310; 14♀♀18♂♂, same data, but 12.VII.1984, Nos. 843372, 843387, 843373, 843381, 843382, 843392, 843429, 843384, 843383, 843363, 843388, 843389, 843390, 843398, 843376, 843380, 843371, 843369, 843364, 843365, 843379, 843366, 843374, 843385, 843386, 843368, 843367, 843375, 843393, 843394, 843395, 843397; 6♀♀4♂♂, same data, but 13.VII.1984, Nos. 843542, 843553, 843537, 843538, 843547, 843546, 843536, 843551, 843543, 843549, 843550; 2♀♀2♂♂, same data, but 16.VII.1984, Nos. 843670, 843669, 843666, 843672; 3♀♀6♂♂, same data, but 18.VII.1984, Nos. 843752, 843754, 843746, 843748, 843753, 843747, 843744, 843745, 843749; 3♀♀, same data, but 19.VII.1984, Nos. 843767, 843769, 843771; 1♀, same data, but 29.VII.2007, Wang Yiping; 2♀♀4♂♂, Longquan Fengyangshan Fengyangjian, 27.VII.2007, Liu Jinxian, Nos. 200801320, 200801343, 200801347, 200801348, 200801349, 200801350; 1♀, same data, but 30.VII.2007, No. 200802856; 1♀1♂, Qingyuan Baishanzu, 27.V.1993, Wu Hong, Nos. 946490, 946495; 3♀♀, same data, but 21.VIII.1993, Nos. 940639, 940640, 940641; 1♀, same data, but 18.VII.1994, No. 9406817; 1♀, Xitianmushan Xianrending, 27.VII.1998, Zhao Mingshui, No. 993045; 1♀, same data, but 16.VIII.1998, Chen Xuexin, No. 997286. Fujian prov.: 1♀, Dazhulan, 29.VII.1983, Wang Jiashe, No. 854446; 1♀, same data, but 15.VII.1994, Chen Xuexin, No. 941935; 1♂, Wuyishan Huanggangshan, 14.VII.1983, Liu Minghui; 1♀, Wuyishan Tongmu, 14.VII.1994, Cai Ping, No. 943444. Shaanxi prov.: 1♀, Qinling Tiantaishan, 3.IX.1999, Chen Xuexin, No. 991274. Sichuan prov.: 6♀♀13♂♂, Wolong, 20.VII.2006, Wang Yiping; 1♂, Wolong, 21.VII.2006, Wang Yiping. Henan prov.: 1♀, Baotianman, 13–15.VII.1998, Ma Yun, No. 987472; 1♀, same data, but 15.VII.1998, No. 987194. China (SHEM). Zhejiang prov.: 1♂, Qingyuan Baishanzu, 24.VII.1963, Jin Gentao, No. 34021437. Fujian prov.: 1♀, Fengyangshan, 24.VI.1932, No. 34021479. China (RMNH). Zhejiang prov.: 1♀, Fengyangshan, 11.VII.1984, Shen Lirong, No. 843304; 3♀♀1♂, same data, but 12.VII.1984, Nos. 843370, 843377, 843391, 843378; 1♂, same data, but 18.VII.1984, No. 843750.

**Figures 1–9.**
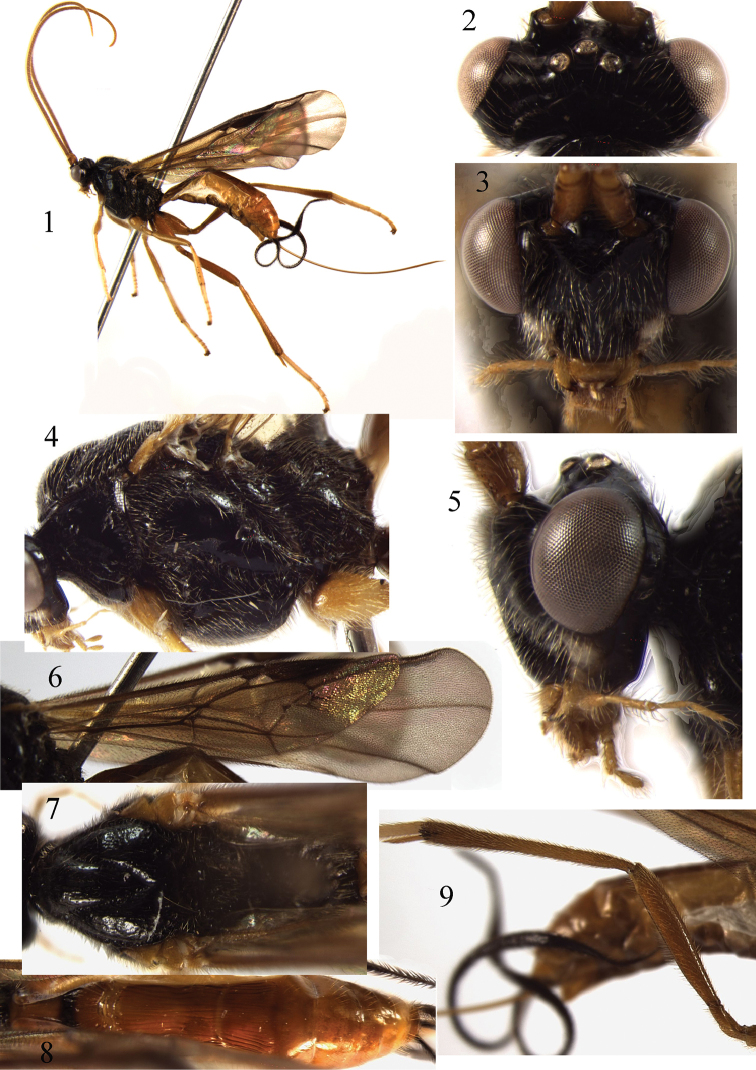
*Braunsia antefurcalis* Watanabe, 1937. ♀, China. **1** habitus, lateral aspect **2** head, dorsal aspect **3** head, front aspect **4** mesosoma, lateral aspect **5** head, lateral aspect **6** fore wing **7** mesosoma, dorsal aspect **8** metasoma, dorsal aspect **9** hind femur and tibia.

##### Remarks.

*Braunsia antefurcalis* is very similar to *B. guangdong* sp. n. and *B. longicoxa*. The differences between them are in the lengths of the first tergite, second tergite, and hind femur as well as the colour pattern in face, clypeus, and wing membranes.

##### Distribution.

Oriental and East Palaearctic regions. China (Zhejiang, Fujian, Henan, Sichuan, Shaanxi); Japan; Russia (Yu et al. 2017).

#### Braunsia bipunctata

Taxon classificationAnimaliaHymenopteraBraconidae

Enderlein, 1906

Braunsia bipunctata Enderlein, 1906: 263; Shenefelt 1970: 371; Bhat and Gupta 1977: 64; Chou and Sharkey 1989: 175; Chen and Yang 2006: 105.

##### Remarks.

Chou and Sharkey (1989) and Chen and Yang (2006) recorded this Indonesian species from Taiwan and Fujian, respectively, but these specimens may be misidentified. According to the description by Chou and Sharkey (1989) and Chen and Yang (2006) their specimens differ from the Indonesian *B. bipunctata* by having a complete and regular basal transverse carina on the propodeum (transverse propodeal carina partly weakly developed and irregular in *B. bipunctata*) and the large stigmal spot of the fore wing connected to a dark brown band below it (without dark band below stigmal spot in *B. bipunctata*). Unfortunately, we did not have access to these specimens and their taxonomic position remains uncertain.

##### Distribution.

Oriental region. China (Fujian?, Taiwan?); Indonesia (Yu et al. 2017).

#### Braunsia fumipennis

Taxon classificationAnimaliaHymenopteraBraconidae

(Cameron, 1899)

Figs 10–18

Microdus fumipennis Cameron, 1899: 96.
Disophrys fumipennis : Dover 1925: 40.
Bassus fumipennis : Thompson 1953: 94.
Braunsia fumipennis : Baltazar 1963: 2; Shenefelt 1970: 373; Bhat and Gupta 1977: 69; Sharkey and Clutts 2011: 87.
Braunsia pumatica van Achterberg & Long, 2010: 45 (syn. by Sharkey and Clutts 2011).

##### Material examined.

Vietnam (RMNH). Holotype of *B. pumatica*, ♀, “S. Vietnam: Dak Lak, Chu Yang Sin N.P. Krong K’Mar, Mal. traps 740–900 m, 2–10.vii.2007, C. v. Achterberg & R. de Vries, RMNH’07”. China (ZJUH). Yunnan prov.: 1♂, Xishuangbanna, 30.VII.2003, Xu Zaifu, No. 20055461. China (SHEM). Tibet: 1♀, Motuo Kabu, 7.V.1980, Jin Gentao & Wu Jianyi, No. 34201571.

**Figures 10–18.**
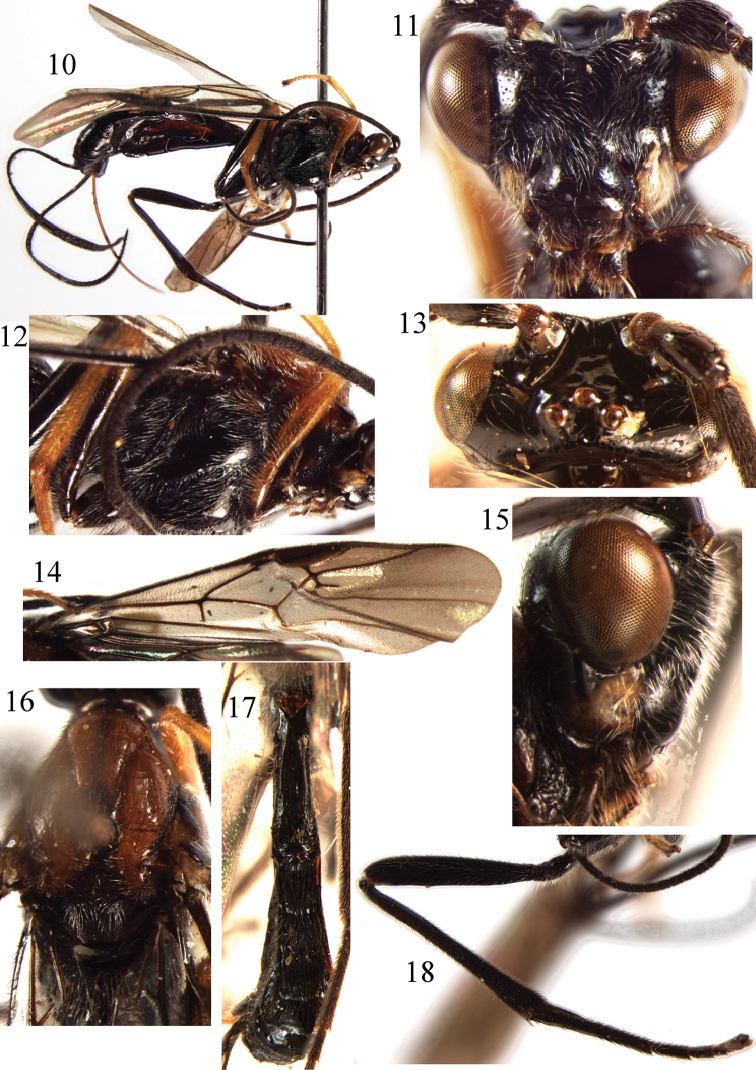
*Braunsia fumipennis* (Cameron, 1899). ♀, China. **10** habitus, lateral aspect **11** head, front aspect **12** mesosoma, lateral aspect **13** head, dorsal aspect **14** fore wing **15** head, lateral aspect **16** mesosoma, dorsal aspect **17** metasoma, dorsal aspect **18** hind leg.

##### Remarks.

*Braunsia fumipennis* is similar to *B. pilosa*, but differs in the the body colour pattern (hind tibia brown; mesosoma black); shorter length of first tergite (2.3 times its apical width); and apical half of first tergite smooth.

##### Distribution.

Oriental region. China (Yunnan, Tibet) new record; India; Myanmar; Thailand; Vietnam (Yu et al. 2017).

#### Braunsia guangdongensis sp. n.

Taxon classificationAnimaliaHymenopteraBraconidae

http://zoobank.org/B331A16C-C0B0-4BB9-89E3-34C467998D0C

Figs 19–27

##### Material examined.

Holotype. ♀, Guangdong prov., Longmen Nankunshan, 14–15.VII.2003, Xu Zaifu, No. 20053640 (ZJUH). Paratypes: 2♂♂, same data, but No. 20053619, 20053641 (ZJUH); 1♀, Guangdong prov., Ruyuan Nanling, 23.VII.2003, Xu Zaifu, No. 20049043 (ZJUH); 1♀, same data, but 18.VII.2003, No. 20049357 (ZJUH); 2♀♀, Guangdong prov., Meizhou Fengxi, 29.VII.2003, Chen Jujian, No. 20048502, 20048677 (ZJUH).

**Figures 19–27.**
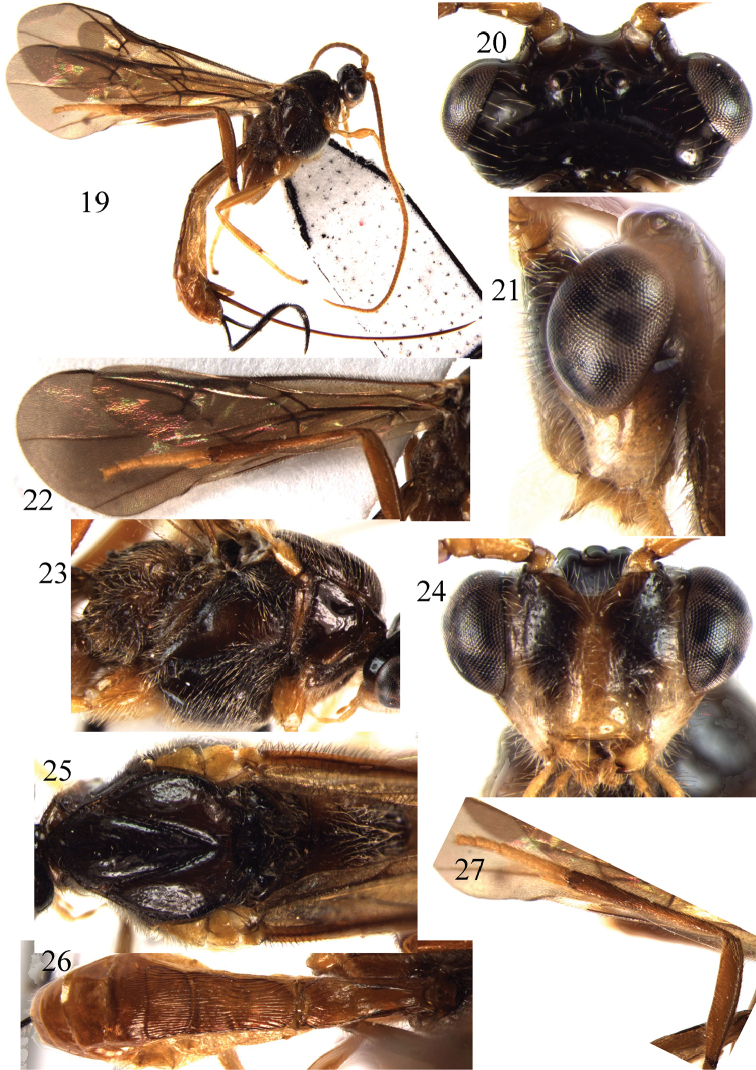
*Braunsia guangdongensis* sp. n., ♀, holotype. **19** habitus, lateral aspect **20** head, dorsal aspect **21** head, lateral aspect **22** fore wing **23** mesosoma, lateral aspect **24** head, front aspect **25** mesosoma, dorsal aspect **26** metasoma, dorsal aspect **27** hind leg.

##### Diagnosis.

Body black. Antenna, hind coxa and hind femur yellowish brown. Area below face and clypeus ivory. Wing membrane evenly dark brown. Vein cu-a of fore wing antefurcal. Length of hind femur 5.2–5.3 times as long as wide. Length of first tergite 2.7–2.8 times its apical width. Ovipositor sheath ribbon-shaped widened.

##### Description.

Holotype, ♀, length of body 9.0 mm, of fore wing 7.0 mm.

*Head*. Antennal segments 45, length of third segment 1.15 times fourth segment, length of third, fourth and penultimate segments 2.5, 2.2 and 1.7 times their width, respectively; length of maxillary palp 0.7 times height of head; in dorsal view head transverse and 1.3 times as wide as mesoscutum; length of eye 2.2 times temple; POL:OD:OOL = 9:6:13; antennal sockets not tubular; occipital flange sharp; malar space 1.8 times as long as basal width of mandible; face shiny with sparse fine punctures, frons and vertex smooth.

*Mesosoma*. Length of mesosoma 1.5 times its height; subpronope large and deep; side of pronotum smooth; area near lateral carina of mesoscutum crenulate; lateral lobes of mesoscutum almost smooth, sparsely finely punctate anteriorly; middle lobe with sparse fine punctures; notauli deep, smooth; scutellar sulcus 0.5 times as long as dorsal face of scutellum and with one carina; scutellum convex anteriorly, smooth and with long setae; mesopleuron above precoxal sulcus largely smooth; mesopleuron below precoxal sulcus setose, with sparse fine punctures; precoxal sulcus wide, shallow and distinctly crenulate; metapleuron mainly smooth with long setae; propodeum setose, with a strong transverse carina subbasally, rugose posteriorly; spiracle medium-sized, round, 1.8 times as long as wide.

*Wings*. Fore wing: second submarginal cell pentagonal, narrow anteriorly, with rather long ramellus, 0.9 times as long as vein 2-SR (14:15); r:3-SR:SR1 = 8:3:72; 2-SR:3-SR:r-m = 15:3:15; vein cu-a antefurcal. Hind wing: vein 2-SR+M transverse; vein M+CU 0.5 times as long as 1-M; surroundings of cu-a glabrous.

*Legs*. Length of hind femur, tibia and basitarsus 5.2, 9.2 and 5.0 times their width, respectively; hind coxa smooth; hind femur with short and sparse setosity; outer side of apical third of middle tibia with a row of 4 pegs; outer side of apex of hind tibia with a cluster of 6 pegs; length of outer and inner spurs of middle tibia 0.4 and 0.5 times middle basitarsus, respectively; length of outer and inner spurs of hind tibia 0.3 and 0.4 times hind basitarsus.

*Metasoma*. First tergite slender shiny, rugulose near apex, slightly and roundly widened apically; length of first tergite 2.7 times its apical width; dorsal carinae of first tergite divergent and on three-fourths of tergite; second tergite 1.6 times as long as wide apically and with posteriorly diverging striae, apical third of second tergite with transverse furrow; anterior half of third tergite striate and apical half finely granulate; remainder of metasoma smooth, ovipositor sheath wide and ribbon-shaped, as long as fore wing.

*Colour*. Black; malar space, lower part of temple and face laterally narrowly ivory, clypeus, palpi and medial part of face pale yellow; antenna, legs and metasoma yellowish-brown, but tarsi paler than tibiae; wing membrane rather dark brown.

**Male.** Unknown.

##### Variations.

Vein M+CU of hind wing 0.5–0.6 times as long as 1-M; length of first tergite 2.7–2.8 times its apical width; length of hind femur, tibia and basitarsus 5.2–5.3, 9.0–9.4 and 5.0–5.2 times their width; outer side of apical third of middle tibia with a row of 3–5 pegs; outer side of hind tibial apex with cluster of 67 pegs.

##### Distribution.

Oriental region. China (Guangdong).

##### Biology.

Unknown.

##### Remarks.

This new species is very similar to *B. antefurcalis* Watanabe, but differs in having the first tergite 2.7–2.8 times as long as its apical width; length of hind femur about 5.2–5.3 times as long as its width and area below face and clypeus ivory.

##### Etymology.

From “Guangdong”, the province of the type locality.

#### Braunsia longicoxa

Taxon classificationAnimaliaHymenopteraBraconidae

Bhat & Gupta, 1977

Figs 28–34

Braunsia longicoxa Bhat & Gupta, 1977: 74; Chou and Sharkey 1989: 176; Chen and Yang 2006: 106.

##### Material examined.

China (ZJUH). Guangxi prov.: 1♀, Longsheng Huaping, 25–26.VI.1982, He Junhua, No. 823503. Hainan prov.: 1♀, Jianfengling Tianchi, 22–23.X.2007, Liu Jingxian, No. 200710767. China (CAU). Guangxi prov.: 1♂, Huaping Hongtan, 12.VI.1963, Yang Jikun.

**Figures 28–34.**
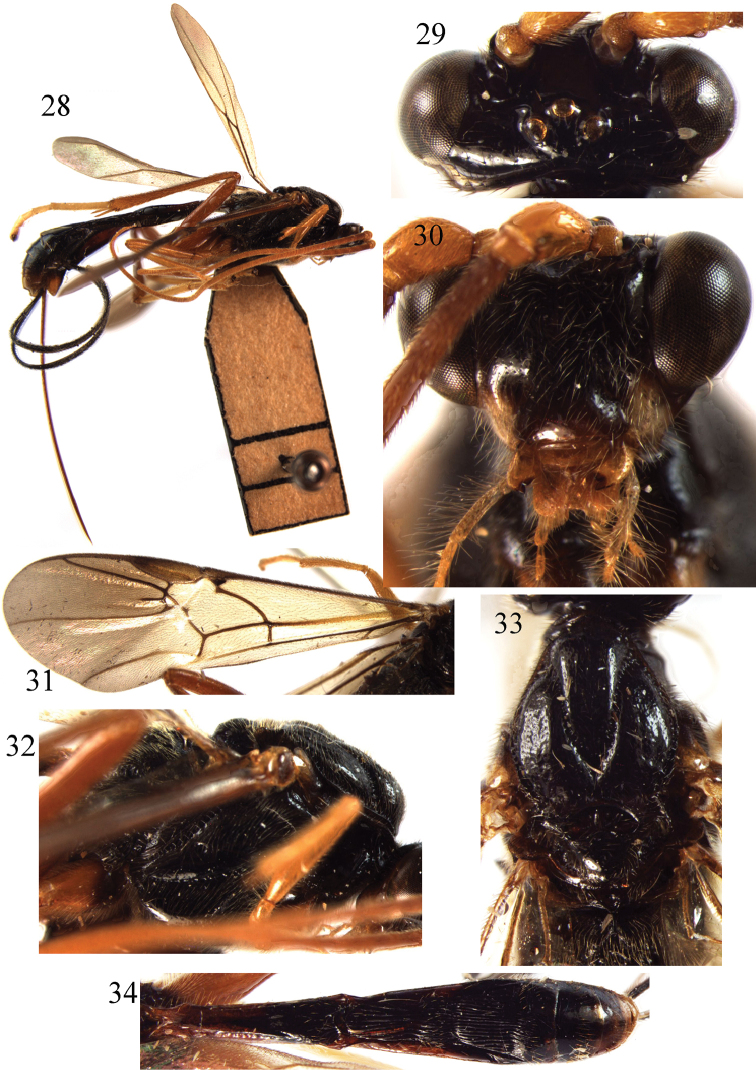
*Braunsia longicoxa* Bhat & Gupta, 1977. ♀, China. **28** habitus, lateral aspect **29** head, dorsal aspect **30** head, front aspect **31** fore wing **32** mesosoma, lateral aspect **33** mesosoma, dorsal aspect **34** metasoma, dorsal aspect.

##### Remarks.

Similar body colour pattern to *B. antefurcalis* and *B. guangdong* sp. n., but differs in having long first and second tergites; colour pattern in wing membrane (only dark brown in apical half).

##### Distribution.

Oriental region. China (Guangxi, Hainan, Taiwan); Philippines (Yu et al. 2017).

#### Braunsia matsumurai

Taxon classificationAnimaliaHymenopteraBraconidae

Watanabe, 1937

Figs 35–41

Braunsia matsumurai Watanabe, 1937: 89; Shenefelt 1970: 373; Belokobylskij 1989: 62; Sharkey 1996: 60; He et al. 2001: 373.

##### Material examined.

China (ZJUH). Zhejiang prov.: 1♀, Anji Longwangshan, 31.VIII.1993, Ma Yun, No. 9310355; 2♀, same data but Chen Xuexin, No. 939821, No. 9310700; 2♂♂, Linhai, 19.V.1935. 1♀, Xitianmushan, 5.VI.1989, He Junhua, No. 890810; 1♂, 12.VI.1933. Fujian Prov.: 1♀, Wuyishan, 13.VII.1986, Wang Jiashe, No. 865590; 1♀, Wuyishan Dazhulan, 31.VII.1983, Ma Yun, No. 833095. Hunan Prov.: 1♀, Daoxian, 31.VII.1982, Tong Xinwang, No. 846381. Guangdong Prov.: 1♀, Nankunshan, 8.VI.2002, Xu Zaifu, No. 20028808; 1♂, Ruyuan Nanling, 23.VII.2003, Xu Zaifu, No. 20049058. Guangxi Prov.: 1♀, Longsheng Huaping Tianpingshan, 22.VI.1982, He Junhua, No. 823257; 1♀, Longzhou Nonggang, 18.V.1982, He Junhua, No. 821478. China (SHEM). Fujian Prov.: 1♀1♂, Guangze Siqian, 30.IV.1960, Jin Gentao & Lin Yangming, Nos. 34021494, 34021495; 1♀, Wuyishan, 18.VII.1985, Jin Gentao, No. 34013761; 1♀, Jianning Jinraoshan, 11.VII.1959, Jin Gentao & Lin Yangming, No. 34021285. Zhejiang Prov.: 1♀, Tianmushan, 11.VI.1936, O. Piel, No. 34021444; 1♀, Taishun, 27.VI.1963, Jin Gentao, No. 34021391. China (RMNH). Zhejiang Prov.: 1♀, Anji Longwangshan, 31.VIII.1993, He Junhua, No. 9310703. Hunan Prov.: 1♀, Dayong, 27.VII.1983, Wu Huifang, No. 840657; 1♀, Jiangyong Daboshui, 24.VII.2008, Su Tianming, 25°22.418'N, 111°16.219'E.

**Figures 35–41.**
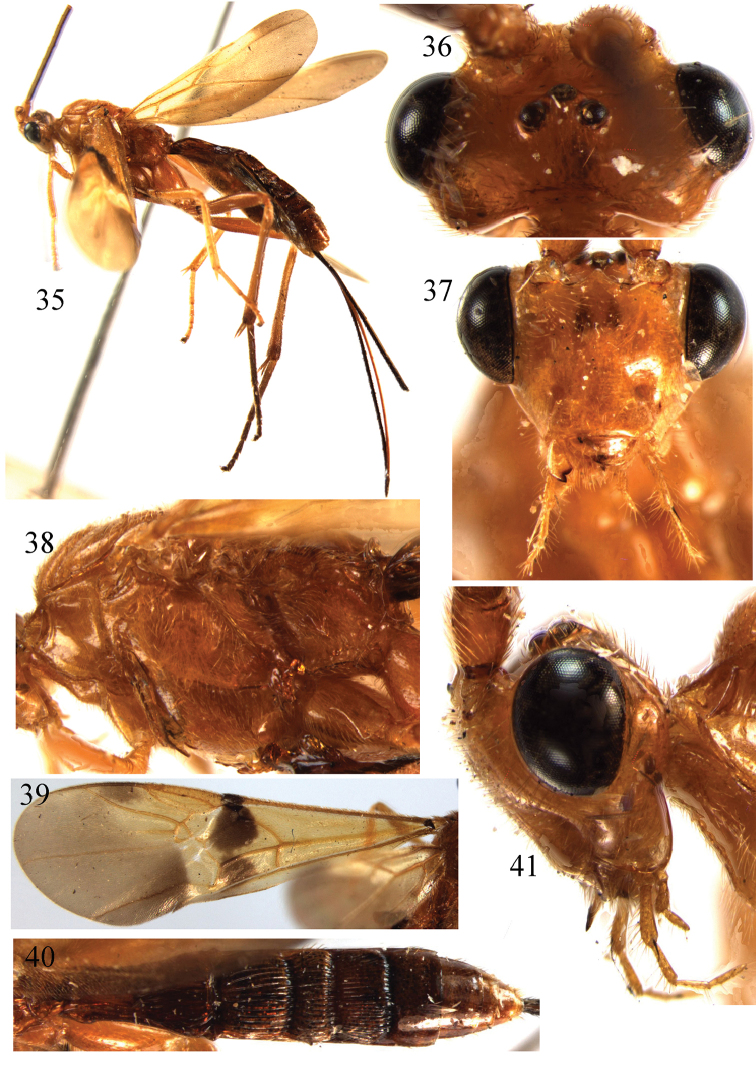
*Braunsia matsumurai* Watanabe, 1937. ♀, China. **35** habitus, lateral aspect **36** head, dorsal aspect **37** head, front aspect **38** mesosoma, lateral aspect **39** fore wing **40** metasoma, dorsal aspect **41** head, lateral aspect.

##### Remarks.

This species is similar to *B. pappi*, especially in the colour pattern, but differs in the basal half of first tergite with distinct striae; hind tibia brownish yellow; tegulae and mesoscutum with same colour (tegulae whitish yellow, contrasting with brownish yellow mesoscutum in *B. pappi*).

##### Distribution.

Oriental and East Palaearctic regions. China (Zhejiang, Fujian, Hunan, Guangdong, Guangxi); Japan; Korea (Yu et al. 2017).

#### Braunsia pappi

Taxon classificationAnimaliaHymenopteraBraconidae

Chen & Yang, 2006

Braunsia pappi Chen & Yang, 2006: 107.

##### Remarks.

This species is only recorded from China (Fujian). It is similar to *B. matsumurai*, and see the differences between them in the diagnosis of *B. matsumurai*. The illustrations of *B. pappi* provided by Sharkey and Yu clearly show that the length of first tergite is actually 1.8 times as long as its apical width not 3.0 times as mentioned in the description of Chen and Yang (2006).

##### Distribution.

Oriental region. China (Fujian) (Yu et al. 2017).

#### Braunsia pilosa

Taxon classificationAnimaliaHymenopteraBraconidae

Belokobylskij, 1986

Figs 42–50

Braunsia pilosa Belokobylskij, 1986: 33; 1989: 64; Sharkey 1996: 61.

##### Material examined.

China (ZJUH). Henan Prov.: 1♀, Songxian Baiyunshan, 19.VII.1996, Cai Ping, No. 985703. Yunnan Prov.: 1♀, Chuxiong, 18.IX.1981, Li Fasheng, No. 200012392. Zhejiang Prov.: 1♀, Tianmushan, 21.VII.1936, O. Piel; 1♀, Xitianmushan, 21.VII.1937. China (SHEM). Anhui Prov.: 1♀, Huangshan, 24.VIII.1964, Jin Gentao, No. 34021301. Zhejiang Prov.: 1♀, Xitianmushan, 20.VII.1937, No. 34021450; 1♀, Xitianmushan, 30.VII.1937, No. 34021452.

**Figures 42–50.**
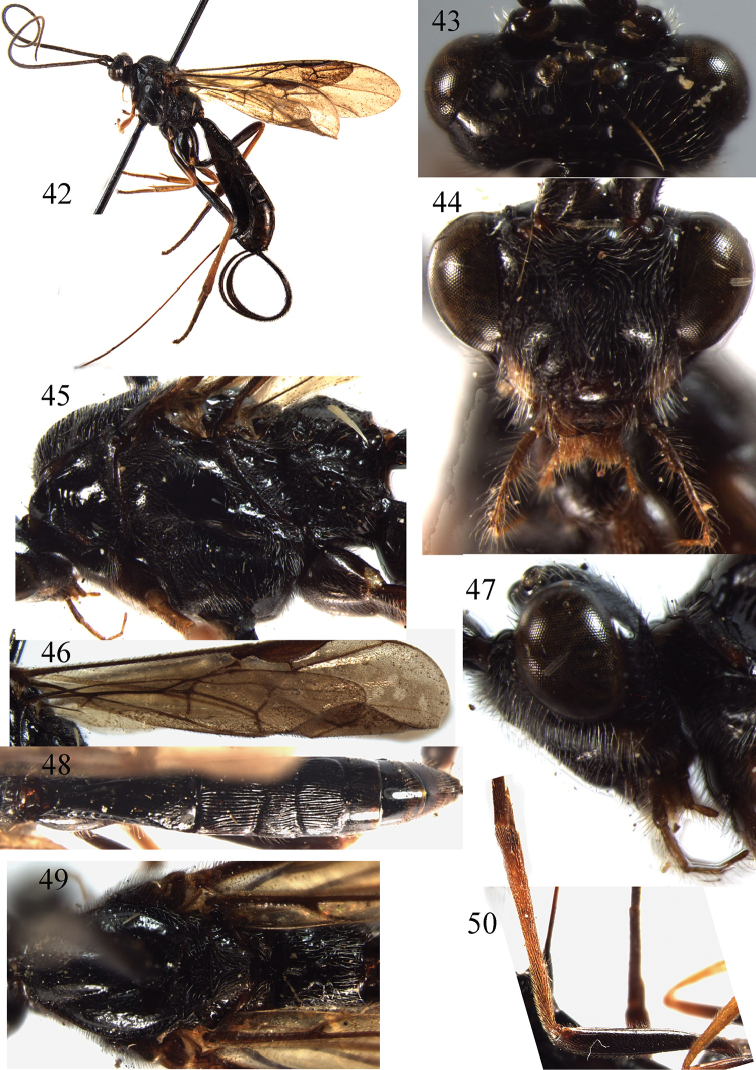
*Braunsia pilosa* Belokobylskij, 1986. ♀, China. **42** habitus, lateral aspect **43** head, dorsal aspect **44** head, front aspect **45** mesosoma, lateral aspect **46** fore wing **47** head, lateral aspect **48** metasoma, dorsal aspect **49** mesosoma, dorsal aspect **50** hind femur and tibia.

##### Remarks.

This species almost melanistic, the wings infuscate; apical half of first tergite smooth; ovipositor sheath ribbon-shaped and widened.

##### Distribution.

Oriental and East Palaearctic regions. China (Henan, Zhejiang, Anhui, Yunnan) **new record**; Japan; Russia (Yu et al. 2017).

#### Braunsia postfurcalis

Taxon classificationAnimaliaHymenopteraBraconidae

Watanabe, 1937

Figs 51–59

Braunsia postfurcalis Watanabe, 1937: 88; Shenefelt 1970: 375; Belokobylskij 1989: 60; Sharkey 1996: 62.

##### Material examined.

China (ZJUH). Zhejiang Prov.: 1♀, Xitianmushan, 3.VIII.1984, Shen Lirong, No. 844646. China (SHEM). Anhui Prov.: 1♀, Huangshan, 26.VIII.1964, Jin Gentao, No. No. 34021299.

**Figures 51–59.**
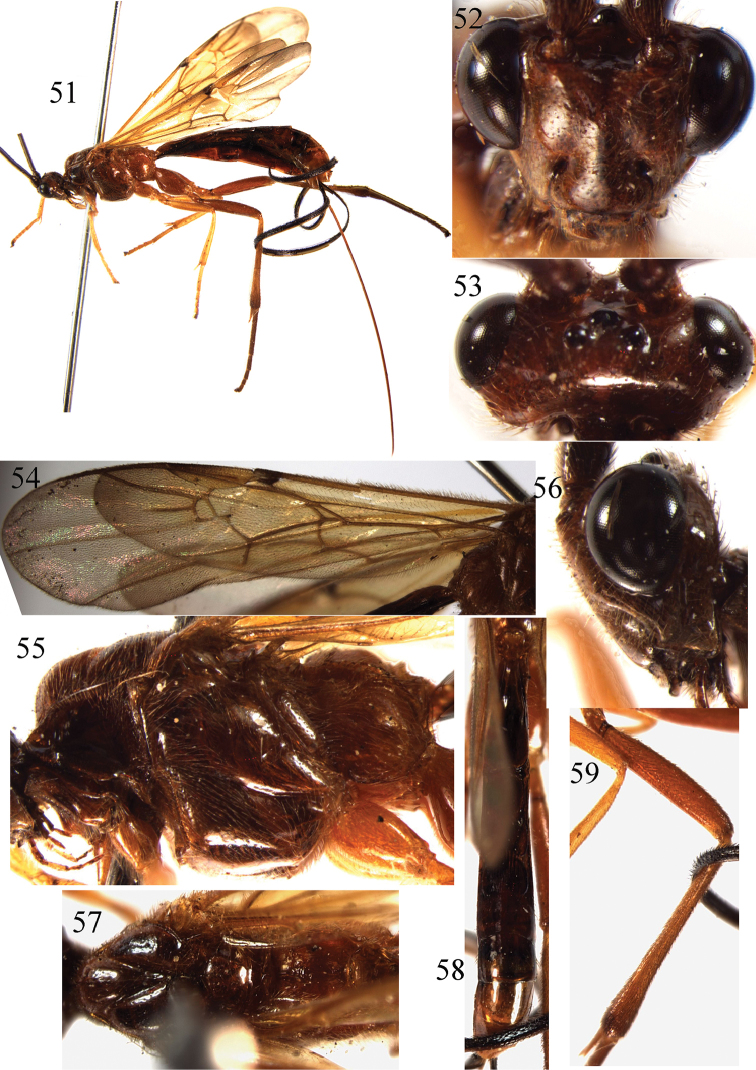
*Braunsia postfurcalis* Watanabe, 1937. ♀, China. **51** habitus, lateral aspect **52** head, front aspect **53** head, dorsal aspect **54** wings **55** mesosoma, lateral aspect **56** head, lateral aspect **57** mesosoma, dorsal aspect **58** metasoma, dorsal aspect **59** hind femur and tiba.

##### Remarks.

This species yellowish brown, the wings and pterostigma yellow; the first tergite is almost entirely smooth; the ovipositor sheath is long, almost as long as body; fore wing without isolated stigma spot.

##### Distribution.

Oriental and East Palaearctic regions. China (Zhejiang, Anhui) new record; Japan (Yu et al. 2017).

#### Braunsia shenyangensis sp. n.

Taxon classificationAnimaliaHymenopteraBraconidae

http://zoobank.org/AEFB0A4F-B8B4-4577-B499-2E86EFE6CD38

Figs 60–68

##### Material examined.

Holotype. ♀, China, Liaoning prov., Shenyang, IX.1955, No. 6503222 (ZJUH).

**Figures 60–68.**
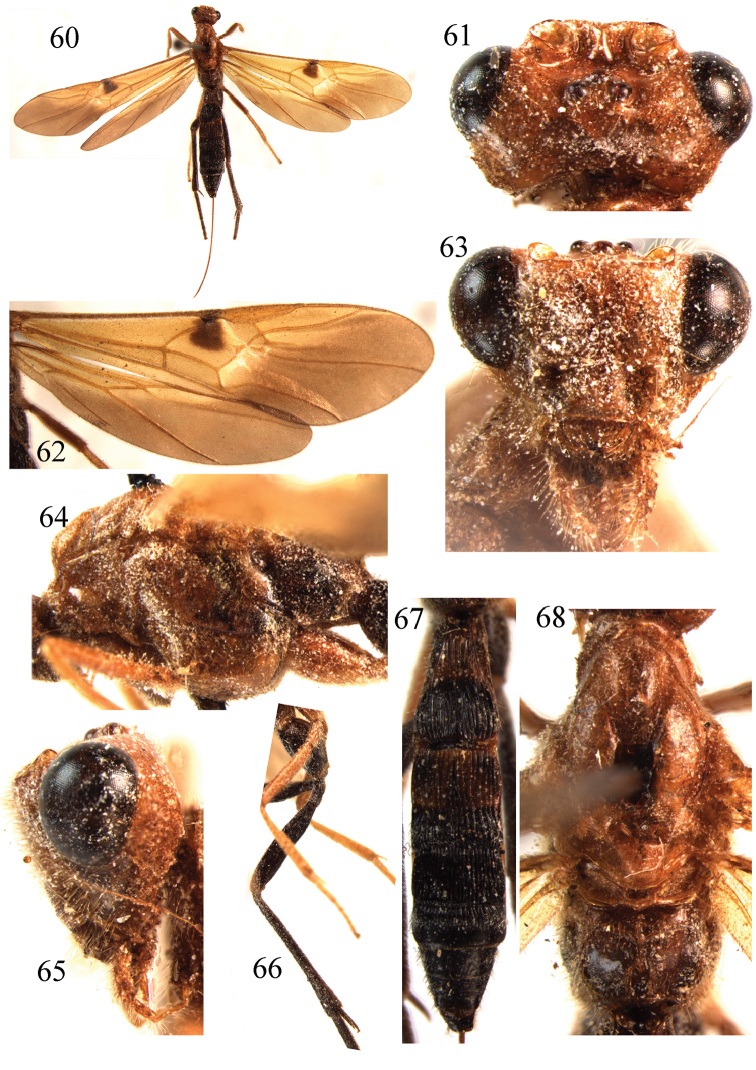
*Braunsia shenyangensis* sp. n., ♀, holotype. **60** habitus, lateral aspect **61** head, dorsal aspect **62** wings **63** head, front aspect **64** mesosoma, lateral aspect **65** head, lateral aspect **66** hind leg **67** metasoma, dorsal aspect **68** mesosoma, dorsal aspect.

##### Diagnosis.

Body brownish yellow. Hind leg black. Fore wing with a small isolated stigmal spot. Pterostigma yellow. Propodeum without a closed areola. Vein cu-a of fore wing almost interstitial; Length of first tergite 1.8 times its apical width. First tergite entirey longitudinally carinate. length of second tergite 1.2 times its width. Ovipositor sheath not widened, distinctly shorter than body.

##### Description.

Holotype, ♀, length of body 15.1 mm, of fore wing 13.5 mm.

*Head*. Antennal segments missing; in dorsal view length of eye twice temple; POL:OD:OOL = 8:6:14; occipital flange large, its ventral margin convex bellow; face shiny smooth with sparse punctures; frons smooth, vertex smooth, sparsely setose.

*Mesosoma*. Length of mesosoma 1.5 times its height; subpronope large and deep; side of pronotum smooth; area near lateral carina of mesoscutum smooth; lateral lobes of mesoscutum almost smooth; notauli deep, smooth, scutellar sulcus 0.5 times as long as dorsal face of scutellum and with 3 carinae; scutellum smooth, distinctly convex anteriorly and sloping posteriorly; mesopleuron above precoxal sulcus shiny and smooth, below precoxal sulcus shiny with minute punctures; precoxal sulcus narrow, similar to a smooth groove; metapleuron smooth; propodeum with a subbasal transverse carina, without a closed areola, spiracle large, elliptical, close to lateral carina and 2.8 times as long as wide; lateral carina of propodeum completely.

*Wings*. Fore wing: second submarginal cell pentagonal, narrow anteriorly, with rather long ramellus, 1.4 times as long as vein 2-SR (14:10); r:3-SR:SR1 = 9:4:70; 2-SR:3-SR:r-m = 14:4:14; vein cu-a almost interstitial. Hind wing: vein 2-SR+M transverse; vein M+CU 0.9 times as long as 1-M; surroundings of vein cu-a sparsely setose.

*Legs*. Length of hind femur, tibia and basitarsus 4.9, 8.3 and 8.6 times their width, respectively; hind coxa smooth; hind femur with short and dense setosity; outer side of apical third of middle tibia with a row of 4 pegs and cluster of 4 pegs at apex; outer side of apex of hind tibia with a cluster of 6 pegs; length of outer and inner spurs of middle tibia 0.4 and 0.5 times middle basitarsus, respectively; length of outer and inner spurs of hind tibia 0.3 and 0.4 times hind basitarsus, respectively.

*Metasoma*. First tergite moderately long, widened apically, 1.8 times its apical width; first tergite entirely longitudinally striate; dorsal carinae of first tergite strong, diverging apically; second tergite as long as third tergite, deep striate transverse groove on apical third; third tergite with parallel striae but smooth on extreme apex; striate transverse groove on apical third wide; remainder of metasoma smooth with sparse setae apically; ovipositor sheath broken; ovipositor about as long as fore wing.

*Colour*. Brownish yellow; fore wing with a brown stigmal spot; apical third of wings infuscate and basal two-thirds yellow; parastigma yellow; hind leg black; metasoma black, but basal half of first and second tergites and ventral part of first-third metasomal segments brownish yellow.

**Male.** Unknown.

##### Distribution.

East Palaearctic region. China (Liaoning).

##### Biology.

Unknown.

##### Remarks.

This new species is very similar to *B. matsumurai* Watanabe, but differs by having no closed areola on the propodeum; the fore wing with a small isolated stigmal spot; the hind leg black; and vein cu-a of the fore wing almost interstitial.

##### Etymology.

From “Shenyang”, the type locality of the species.

#### Braunsia smithii

Taxon classificationAnimaliaHymenopteraBraconidae

(Dalla Torre, 1898)

Agathis flavipennis Smith, 1863: 12 (not Agathis flavipennis Brullé, 1846).
Braunia flavipennis : Shenefelt 1970: 372.
Agathis smithii Dalla Torre, 1898: 143 (replacement name).
Braunsia devriesi van Achterberg & Long, 2010: 36. (syn. by Sharkey and Clutts 2011).

##### Material examined.

Vietnam (RMNH). Holotype of *B. devriesi*, ♀, “N. Vietnam: Viet Tri, n[ea]r Thanh Son, Thuong Cuu, 20°59'E, 105°8'N, 350–400 m, 11–16.x.1999, Malaise traps, R. de Vries, RMNH’99”. China (IZCAS). Yunnan prov.: 1♀, Xishuangbanna, Xiaomengyang, 14.X.1957, Zang Lingchao, No. 1911274; 1♀, Jinggu, 1000 m, 13.V.1957, Panfilov, No. 1911276.

##### Diagnosis.

Body bright brownish-yellow; fore wing with dark brown stigmal spot; wing membrane yellowish; vein cu-a of fore wing distinctly postfurcal.

##### Distribution.

Oriental region. China (Yunnan) new record; Vietnam; Thailand; Malaysia; Indonesia (Sharkey and Clutts 2011).

## Supplementary Material

XML Treatment for Braunsia antefurcalis

XML Treatment for Braunsia bipunctata

XML Treatment for Braunsia fumipennis

XML Treatment for Braunsia guangdongensis

XML Treatment for Braunsia longicoxa

XML Treatment for Braunsia matsumurai

XML Treatment for Braunsia pappi

XML Treatment for Braunsia pilosa

XML Treatment for Braunsia postfurcalis

XML Treatment for Braunsia shenyangensis

XML Treatment for Braunsia smithii
